# Novel compound heterozygous mutations in *OCA2* gene associated with non-syndromic oculocutaneous albinism in a Chinese Han patient: a case report

**DOI:** 10.1186/s12881-019-0850-7

**Published:** 2019-07-25

**Authors:** Hairong Wang, Yang Wan, Yun Yang, Hao Li, Liangwei Mao, Shuyang Gao, Jingjing Xu, Jing Wang

**Affiliations:** 1 Anhui Clinical Laboratories, BGI-Anhui, BGI-Shenzhen, Weisan Road, Fuyang, 236000 China; 2 Department of Obstetrics and Gynecology, Fuyang People’s Hospital, Fuyang, 236000 China; 3 Department of Obstetrics and Gynecology, The Second Affiliated Hospital of Zhengzhou University, Zhengzhou, 450052 China; 4 College of Life Sciences, Hubei University, Wuhan, 430062 China; 5 BGI Genomics, BGI-Shenzhen, Shenzhen, 518083 China; 6 Prenatal diagnosis center, Department of Obstetrics and Gynecology, The First Affiliated Hospital of University of Science and Technology of China, Hefei, 230071 China

**Keywords:** Oculocutaneous albinism, *OCA2*, Non-syndromic, Gross deletion, Targeted NGS

## Abstract

**Background:**

Oculocutaneous albinism (OCA) is a group of rare genetically heterogeneous disorders. The present study aimed to identify the genetic cause of a Chinese Han family with non-syndromic oculocutaneous albinism (OCA).

**Case presentation:**

Here, we report an 11-month-old male proband from a Chinese Han non-consanguineous family, who presented with milky skin, yellow white hair, nystagmus, astigmatism, and hypermetropia. We performed the targeted next-generation sequencing (NGS) on the proband and identified two novel compound heterozygous variants (c.1865 T > C (p.Leu622Pro) and exons 17–21 deletion) in *OCA2* gene associated with OCA type 2 (OCA2, OMIM 203200). Meanwhile, a previously reported heterozygous mutation (c.4805G > A) in *MYO7* gene related with Usher syndrome type 1B was found. The online tools SIFT, PolyPhen-2, and Mutation Taster predicted variant c.1865 T > C was probably damaging. The residue p.Leu622 was in a highly conserved region among species by CLUSTALW. Three-dimensional homology model with I-TASSER indicated that p.Leu622Pro variant disturbed the formation of the α-helix, resulting in a random coil structure. The gross deletion (exons 17–21) in *OCA2* gene has was not been reported previously. These two novel variants in *OCA2* gene were inherited from each parent respectively, after verification by Sanger sequencing and quantitative PCR (qPCR) in the family.

**Conclusions:**

This study indicates the two novel compound heterozygous mutations in *OCA2* gene may be responsible for clinical manifestations of OCA2. It expands the mutation spectrum of *OCA2* gene and is helpful to screen for large deletions with targeted NGS protocol in monogenic disease. It also assists the genetic counselling, carrier screening and personalized healthcare of the disease.

**Electronic supplementary material:**

The online version of this article (10.1186/s12881-019-0850-7) contains supplementary material, which is available to authorized users.

## Background

Oculocutaneous albinism (OCA) is a group of genetically heterogeneous autosomal recessive disorders with the deficiency in melanin synthesis. It is characterized by partial or complete loss of hypopigmented skin, hair, and eyes, and always accompanied with ocular abnormalities, such as nystagmus, reduced visual acuity, photophobia, strabismus, foveal hypoplasia, hypopigmentation of the iris, and color vision impairment [1, 2]. The estimated prevalence of OCA varies extremely among ethnicities, with 1 in 17,000 worldwide, 1 in 18,000 in Chinese Han population [1, 3].

OCA is subdivided into non-syndromic OCA (nsOCA) and syndromic OCA in clinic. The non-syndromic OCA only occurs with clinic symptom typically affecting the skin, hair, and eyes. In contrast, syndromic OCA also affects other parts of the body in addition to the typical clinical phenotype with non-syndromic OCA. For instance, Hermansky–Pudlak Syndrome (HPS) includes oculocutaneous albinism, bleeding problems, and abnormal fat-protein compound storage.

The nsOCA can be broadly classified into several subtypes. At present, seven nsOCA genes have been described: *TYR* (OCA1A and OCA1B), *OCA2* (OCA2), *TYRP1* (OCA3), *SLC45A2* (OCA4), *SLC24A5* (OCA6), *LRMDA* (OCA7), and *MC1R* (Modifier of OCA2) [4]. The characteristics of the nsOCA differ among the different subtypes. OCA1A (OMIM 203100), is the most common and severe type, characterized by a complete loss of pigmentation, which caused by mutations in *TYR* gene. The other ns OCA are less severe, featured with some pigmentation over a lifetime. OCA2 (OMIM 203200) ranks as the second common nsOCA subtype [5, 6], and accounts for almost 30% of OCA cases worldwide [7]. The prevalence of OCA2 also differs widely among different populations. OCA2 patients present a mild to moderate pigmentation in hair, skin, and eyes. It is caused by mutations in *OCA2* gene (formerly called as P gene) which is located on chromosome 15q11.2-q12 spanning about 345 kb of genomic DNA in the region. The *OCA2* gene consists of 24 exons (23 coding) encoding an integral membrane protein which has 12 putative transmembrane domains and contains 838 amino acids [1, 8].

Clinical diagnosis of different OCA subtypes is always indistinguishable because of its overlapping and variable manifestations. The molecular and genetic analyses are helpful for accurate diagnosis, prognosis and genetic counseling. In this study, we described an 11-month-old patient diagnosed with OCA from a Chinese family.

## Case presentation

An 11-month-old male proband is the first child of the non-consanguineous parents from China. He presented with creamy white skin, yellow white hair, accompanied with nystagmus, astigmatism, and hypermetropia. His father was phenotypically normal, his mother presented with yellow hair. We obtained approval from the BGI-Shenzhen ethics committee (No. BGI-IRB 17168). Written informed consent was obtained from the patient’s parents for participation in the present study before collecting peripheral blood. The parents of the proband declined publication of the clinical images.

### Genetic analysis

In order to identify the etiology, targeted NGS was carried out on the proband with the 54 inherited eye disease genes panel, which includes four prevalent nsOCA genes: *TYR, OCA2*, *TYRP1,* and *SLC45A2.* (Additional file 1: Table S1). The total DNA extraction was performed using lymphocyte of peripheral blood by the QIAamp DNA extraction kit (Qiagen, Hilden, Germany), following the manufacturer’s instructions and recommendations. Genomic DNA was fragmented into 200 bp to 300 bp using an ultrasonoscope (Covaris S2, Massachusetts, USA). Then library construction was operated as previously published procedure [9]. The enriched library was sequenced using a HiSeq2500 Analyzers (Illumina, San Diego, CA, USA). The pipeline of bioinformatics analysis was performed to screen the mutations as a previous study [10]. The produced sequenceing paired-end reads (90 bp) were aligned to the reference human genome (GRCh37/hg19) by Burrows Wheeler Aligner (bio-bwa.sourceforge.net). Single-nucleotide variant (SNV) and insertion and deletion (indel) were detected by SOAPsnp software (sourceforge.net/projects/soapsnp/) and the SAMtools (samtools.sourceforge.net) respectively. All SNVs and indels were filtered in the dbSNP, HapMap, 1 K human genome database and in-house database of 100 Chinese controls. Calling copy number variation (CNV) was performed according to a previous paper [11–13]. The deletion was identified by comparing the intra- and inter-sample normalized sequencing depth of each exon. Exons with a depth ratio 0.5 were considered to have heterozygous deletion, in contrast to the given sample. The sequence variants interpretation was conducted based on the guideline of the American College of Medical Genetics (ACMG).

We looked for candidate pathogenic variants in the proband by targeted NGS. The captured targeted region was 238,836 bp, and the coverage of the targeted region was 98.9%. The average sequencing depth of the panel was 204.43-fold, with 95.61% of the targeted bases covered a minimum of 30-fold. Three heterozygous variants were detected in the proband. The first was a missense mutation (c.1865 T > C, p.Leu622Pro) in exon 18 of *OCA2* gene, the second was a gross deletion with exons 17–21 encompassed introns in *OCA2* gene, and the third was a previously reported mutaiton (c.4805G > A, p.Arg1602Gln) in exon 35 of *MYO7A* gene with Usher Syndrome (US) [14], which clinically characterized with deafness and gradual vision loss.

**Fig. 1:**
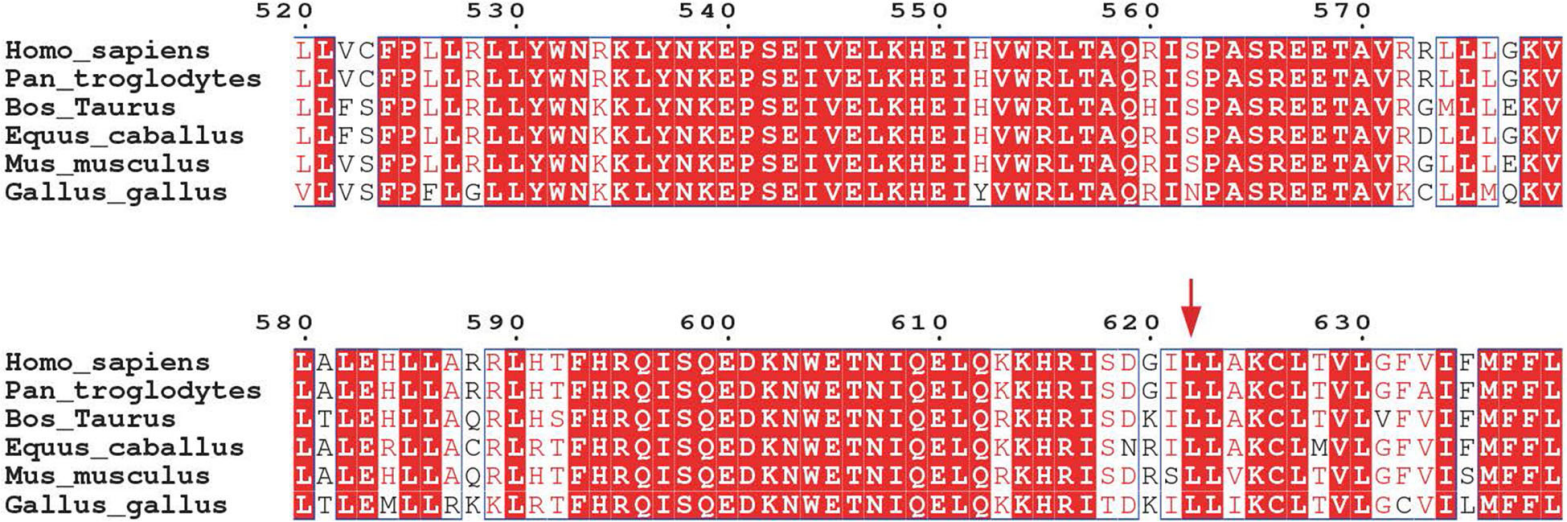
ClustalW alignment of OCA2 protein among representative species around the sites of p.Leu622

**Fig. 2:**
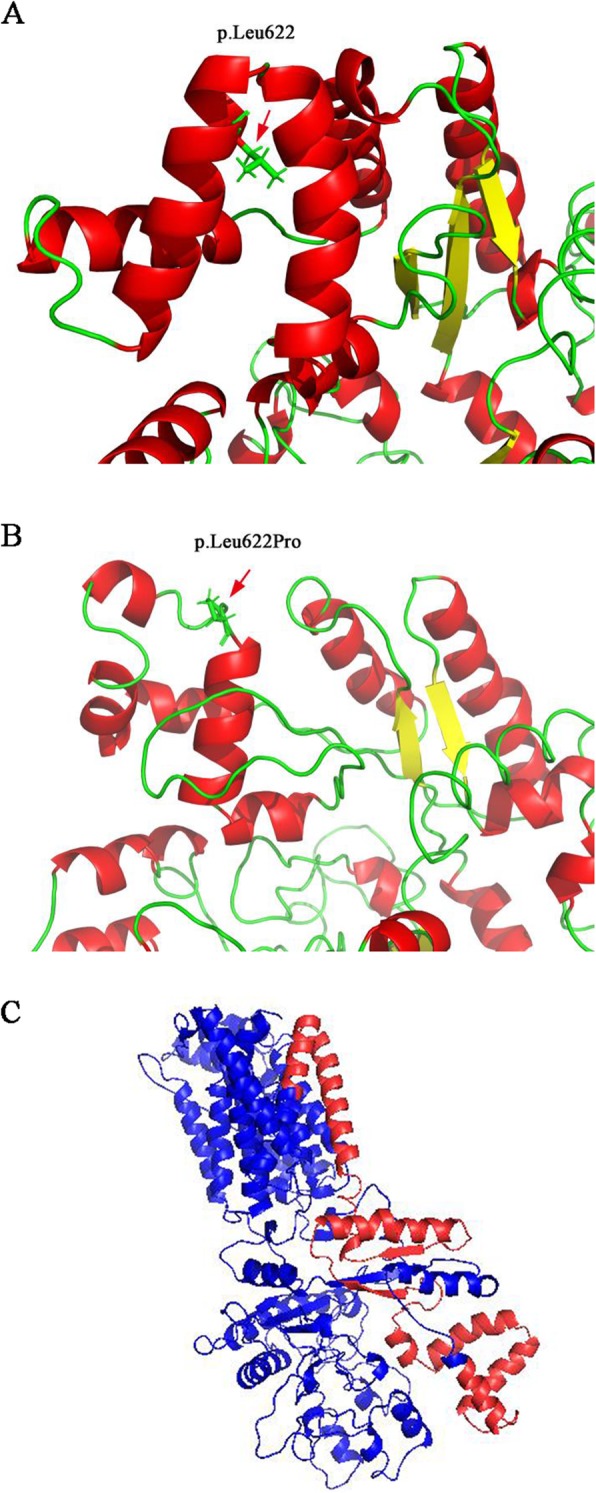
Modeling of the OCA2 protein. **a** p.Leu622. **b** p.Leu622Pro. **c** Structural representation of exons 17–21 deletion. Deleted residues are indicated in red and wild type residues are presented in blue

To further evaluate the pathogenesis of the variant, three prediction programs PolyPhen-2 (http://genetics.bwh.harvard.edu/pph2/), SIFT (http://sift.jcvi.org/), and Mutation Taster (http://www.mutationtaster.org/) were used to predict the probability of variant c.1865 T > C in *OCA2* gene. All prediction tools showed the variant was probably damaging. Multiple amino acid sequence alignment of OCA2 amino acid sequences with CLUSTALW (https://www.genome.jp/tools-bin/clustalw) showed that the missense variant (c.1865 T > C) was in a highly conserved region among species (Fig. 1). The three-dimensional (3D) models of native and mutant proteins were generated to predict the structure of proteins using the I-TASSER (http://zhanglab.ccmb.med.umich.edu/I-TASSER/). The result showed that the variant (c.1865 T > C) disturbed the formation of the α-helix, and formed a random coil structure (Fig. 2a, b), suggesting that it was a pathogenic mutation. Deletion of exons 17–21 affected the 3D structure of OCA2 protein, and leaded to a truncated and non-functional protein (Fig. 2c).

### Confirmation of the novel mutation in the family

**Fig. 3:**
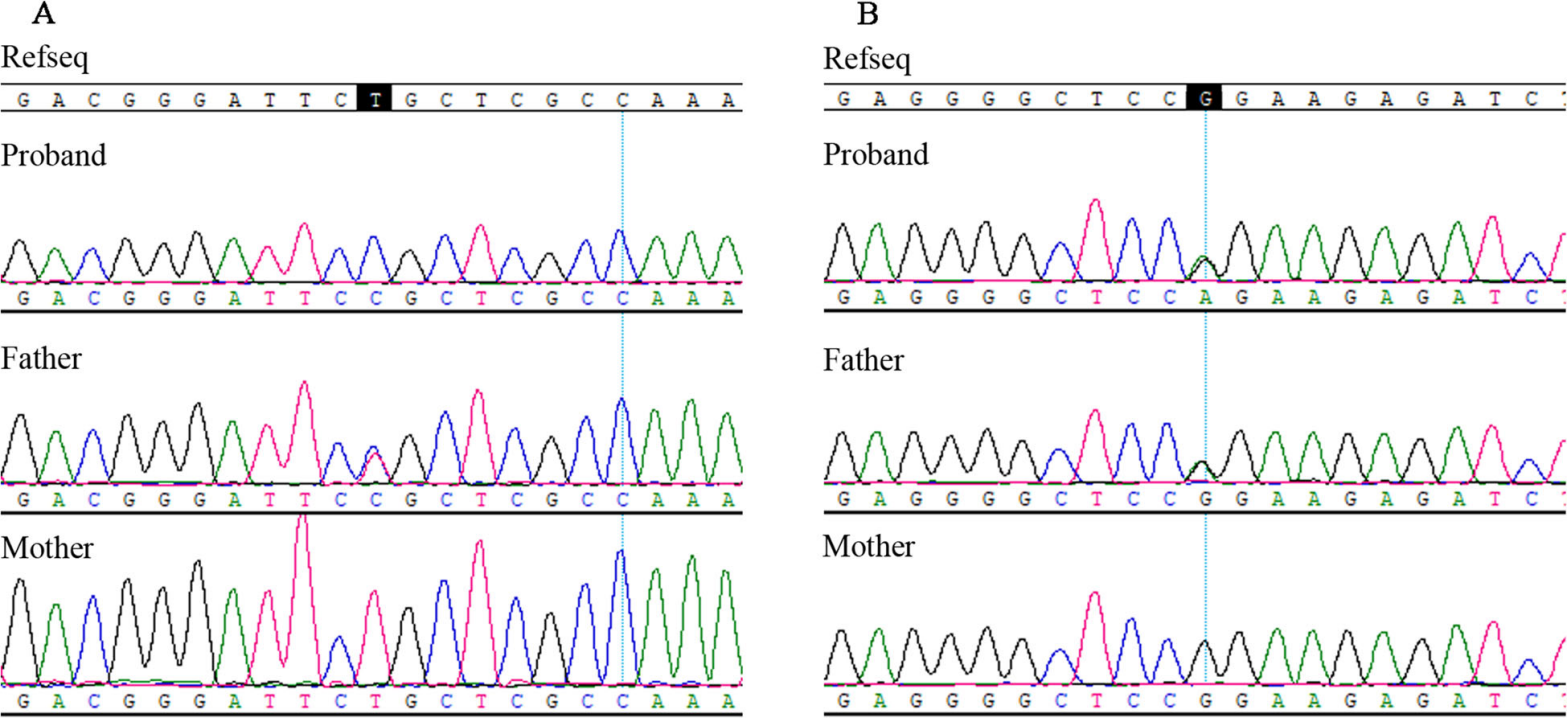
Confirmation of two mutations by Sanger sequencing. **a** OCA2 NM_000275: c.1865 T > C (p.Leu622Pro). **b** MYO7 NM_000260: c.4805G > A (p.Arg1602Gln)

**Fig. 4:**
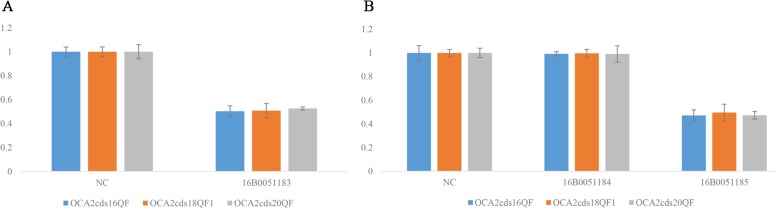
Confirmation of exons 17–21 deletion using qPCR. **a** The qPCR results of the patient. NC represents the control sample, 16B0051183 represents the patient. **b** The qPCR results of the parents. 16B0051184 represents the father of the patient, 16B0051185 represents the mother of the patient

To ascertain three variants revealed by the targeted NGS, we conducted Sanger sequencing and quantitative PCR (qPCR) in the family with primers in Additional file 2: Table S2. The heterozygous variants c.1865 T > C(*OCA2*) and c.4805G > A (*MYO7A*) were both detected in the proband and his father (Fig. 3a, b). The quantity of exon 17–21 detected in the proband was consistent with his mother, almost half of his father and the control sample by qPCR (Fig. 4). The results demonstrated that the compound heterozygous variants in *OCA2* gene of the proband were inherited from the parents respectively, and the proband was also a carrier of US.

## Discussion

The *OCA2* gene encodes integral membrane protein which belongs to the Na+/H+ antiporter family. The polypeptide includes 12 putative transmembrane α-helices domain, encoding an 838 amino acids with 110-KD. It acts as a precursor to melanin synthesis, controls the process of tyrosinase and tyrosinase-related protein [15], also maintains the pH of the melanosomes [16, 17]. The missense mutation c.1865 T > C was previously unreported and predicted to impact the substitution of a proline for the leucine at residue 622 of the homeodomain. The p.Leu622 residue is located within the seventh transmembrane α-helices domain, however, it changes into a random coil strcture in p.Leu622Pro residue and results inthe dysfunction of the OCA2 protein. The mutation p.Leu622Pro is functionally localized in Na-Sulphur-symporter domain which regulates the enzyme activity of tyrosinase by mediating intake of several molecules with the concomitant uptake of Na+ [18]. The deletion of exons 17–21 in *OCA2* gene which spanning from 7 to 10 transmembrane α-helices domains has not been reported before. It causes partial loss of Na-Sulphur-symporter domain, and is predicted to generate non-functional, truncated proteins. Several gross deletions encompassing this region were reported as the genetic cause of the OCA2, such as exons 3–20, exons 1–20, and exons 20–24 deletions in *OCA2* gene [18, 19]. Taking these findings together, these two mutations may be responsible for clinical manifestations of OCA2.

The mutation types causing OCA2 are variable, including single SNV, indel and CNV, and gross deletions. So far, 177 relevant mutations have been reported in HGMD (Human Gene Mutation Database, professional 2019.1). Although the majority was nonsense/ missense mutations, it was notable that 17 gross deletions in *OCA2* gene have been described as genetic lesions for the OCA2 in HGMD. The most common deletion is a 2.7-kb deletion on exon 7 in patients of the African descent [20]. This study sheds light on the importance of routine genetic diagnostics with additional copy number analysis. Compared to Sanger sequencing, the targeted NGS is cost-effective to detect monogenic disorders with various genetic lesions, including SNVs, indels, CNVs, or other factors in one step.

## Conclusions

In summary, we identified two novel compound heterozygous mutations, one missense c.1865 T > C (p.Leu622Pro) and one gross deletion (exons 17–21) in *OCA2* gene in a Chinese Han family. This study enriches the mutation spectrum of OCA. The clinical phenotypes of OCA are always difficult to distinguish, gene diagnosis becomes a useful tool for the precise diagnosis, and assists the genetic counselling, carrier screening and personalized healthcare of the disease.

## Additional files

Additional file 1: The list of 54 genes causing hereditary eye diseases. (DOCX 19 kb)

Additional file 2: Primer sequences for the Sanger sequencing and qPCR. (XLSX 9 kb)

## Data Availability

The datasets reported in this study are deposited on the CNGB Nucleotide Sequence Archive (CNSA: https://db.cngb.org/cnsa; accession number CNP0000188). They are not publicly available due to the ethical principles of BGI-IRB but are available from the corresponding author on reasonable request.

## Abbreviations

3D: Three-dimensional
CNV: Copy number variant; HPS: Hermansky–Pudlak Syndrome
Indel: Insertion/deletion
NGS: Next-generation sequencing
nsOCA: non-syndromic OCA
OCA: Oculocutaneous albinism
OCA1A: Oculocutaneous albinism type 1A
OCA2: Oculocutaneous albinism type 2
qPCR: quantitative PCR
SNV: Single nucleotide variant
US: Usher Syndrome
