# Supplementary material for: Novel compound heterozygous mutations in OCA2 gene associated with non-syndromic oculocutaneous albinism in a Chinese Han patient: a case report
Source: BMC Med Genet. 2019 Jul 25;20:130. doi: 10.1186/s12881-019-0850-7 (PMC6659248; doi:10.1186/s12881-019-0850-7)
Supplement: Supplementary file 1 — The list of 54 genes causing hereditary eye diseases. (DOCX 19 kb) [file 12881_2019_850_MOESM1_ESM.docx]

Table S1. The list of genes analyzed in our study.

| OMIM | Disease | Gene |
| --- | --- | --- |
| 300500 | X-Linked Ocular Albinism | *GPR143* |
| 193510 | Waardenburg syndrome type 2A | *MITF* |
| 203200 | Oculocutaneous Albinism type 2 | *OCA2* |
| 606574 | Oculocutaneous Albinism type 4 | *SLC45A2* |
| 203100 | Oculocutaneous Albinism type 1A | *TYR* |
| 203290 | Oculocutaneous Albinism type 3 | *TYRP1* |
| 203300 | Hermansky-Pudlak Syndrome 1 | *HPS1* |
| 608233 | Hermansky-Pudlak Syndrome 2 | *AP3B1* |
| 614072 | Hermansky-Pudlak Syndrome 3 | *HPS3* |
| 614073 | Hermansky-Pudlak Syndrome 4 | *HPS4* |
| 614074 | Hermansky-Pudlak Syndrome 5 | *HPS5* |
| 614075 | Hermansky-Pudlak Syndrome 6 | *HPS6* |
| 614076 | Hermansky-Pudlak Syndrome 7 | *DTNBP1* |
| 614077 | Hermansky-Pudlak Syndrome 8 | *BLOC1S3* |
| 614171 | Hermansky-Pudlak Syndrome 9 | *BLOC1S6* |
| 209900 | Bardet-Biedl Syndrome 1 | *BBS1* |
| 615981 | Bardet-Biedl Syndrome 2 | *BBS2* |
| 600151 | Bardet-Biedl Syndrome 3 | *ARL6* |
| 615982 | Bardet-Biedl Syndrome 4 | *BBS4* |
| 615983 | Bardet-Biedl Syndrome 5 | *BBS5* |
| 605231 | Bardet-Biedl Syndrome 6 | *MKKS* |
| 615984 | Bardet-Biedl Syndrome 7 | *BBS7* |
| 615985 | Bardet-Biedl Syndrome 8 | *TTC8* |
| 615986 | Bardet-Biedl Syndrome 9 | *BBS9* |
| 615987 | Bardet-Biedl Syndrome 10 | *BBS10* |
| 615988 | Bardet-Biedl Syndrome 11 | *TRIM32* |
| 615989 | Bardet-Biedl Syndrome 12 | *BBS12* |
| 615990 | Bardet-Biedl Syndrome 13 | *MKS1* |
| 615991 | Bardet-Biedl Syndrome 14 | *CEP290* |
| 256100 | Nephronophthisis 1 | *NPHP1* |
| 606966 | Nephronophthisis 4 | *NPHP4* |
| 609254 | Senior-Loken syndrome 5 | *IQCB1* |
| 613615 | Senior-Loken Syndrome 7 | *SDCCAG8* |
| 601386 | Autosomal Recessive Deafness 12 | *CDH23* |
| 602092 | Autosomal Recessive Deafness 18A | *USH1C* |
| 609533 | Autosomal Recessive Deafness 23 | *PCDH15* |
| 606943 | Usher Syndrome Type IG | *USH1G* |
| 276901 | Usher Syndrome Type IIA | *USH2A* |
| 605472 | Usher syndrome, type IIC, GPR98/PDZD7 digenic | *PDZD7、GPR98* |
| 276900 | Usher syndrome, type 1B | *MYO7A* |
| 611383 | Usher syndrome, type 2D | *DFNB31* |
| 614180 | Retinitis pigmentosa 61 | *CLRN1* |
| 610156 | Mental Retardation, Truncal Obesity, Retinal Dystrophy, And Micropenis syndrome | *INPP5E* |
| 608091 | Joubert Syndrome 2 | *TMEM216* |
| 608629 | Joubert Syndrome 3 | *AHI1* |
| 610688 | Joubert Syndrome 6 | *TMEM67* |
| 611560 | Joubert Syndrome 7 | *RPGRIP1L* |
| 612291 | Joubert Syndrome 8 | *ARL13B* |
| 612285 | Joubert Syndrome 9 | *CC2D2A* |
| 300804 | Joubert Syndrome 10 | *OFD1* |
| 200990 | Acrocallosal Syndrome | *KIF7* |
| 600638 | Congenital Fibrosis of the Extraocular Muscles 3A | *TUBB3* |
| 135700 | Congenital Fibrosis of the Extraocular Muscles 1 | *KIF21A* |
